# Continuous Serratus Anterior Versus Erector Spinae Plane Block Catheters for Postoperative Pain Management Following Video-Assisted Thoracoscopic Surgery: A Retrospective Study

**DOI:** 10.7759/cureus.69354

**Published:** 2024-09-13

**Authors:** Carla L Maffeo-Mitchell, Katherine Davis, Sarah Vincze, Edmund T Takata, Ya-Huei Li, Aseel Walker, Ilene Staff, Kevin Finkel

**Affiliations:** 1 Integrated Anesthesia Associates, Hartford Hospital, Hartford, USA; 2 Research, Hartford Hospital, Hartford, USA

**Keywords:** erector spinae plane block, multimodal pain management, regional anesthesia, serratus anterior plane block, video-assisted thoracoscopic surgery (vats)

## Abstract

Introduction: Optimal pain management following video-assisted thoracoscopic surgery (VATS) is key to promoting efficient recovery while minimizing the incidence of postoperative complications. Regional anesthesia can help achieve greater pain control, fostering enhanced recovery and increased patient satisfaction, though debate remains over the most effective technique for VATS. This study sought to compare the analgesic efficacy of two continuous regional anesthetic techniques commonly used for VATS, the serratus anterior plane block (SAPB or CSAPB) and the erector spinae plane block (ESPB or CESPB). This study also sought to identify the clinical benefits of regional anesthesia (CSAPB or CESPB) versus no regional anesthesia in the setting of VATS.

Methods: A retrospective study was conducted, including 397 adult patients who underwent VATS and received multimodal analgesia. Postoperative outcomes were compared among patients who received CSAPB versus those who received CESPB; these outcomes were also compared between patients who received either regional anesthesia technique (CSAPB or CESPB, block group) and patients who did not receive regional anesthesia (non-block group). Co-primary outcomes included opioid consumption during hospital admission (presented as morphine milligram equivalents) and pain (minimum, maximum, and average numeric pain scale scores) in the first 72 postoperative hours. Secondary postoperative outcomes included post-anesthesia care unit (PACU) length of stay, time from procedure end to discharge, time to first opioid medication, ambulation distance on day one, medication use, and incidence of surgical or block-related complications. All data were retrospectively obtained from patients' electronic medical records.

Results: Comparing regional anesthesia techniques, patients who received CESPB reported lower pain with activity postoperatively than patients who received CSAPB (3.6 vs. 4.2, p=0.009). There were no other significant differences in postoperative outcomes between these groups. Comparing the block and non-block groups, the block group exhibited a higher overall comorbidity burden than the non-block group (p=0.001). Even so, the block group reported less postoperative pain at rest and with activity than the non-block group (mean: 3.6 vs. 4.1, p=0.012; mean 3.8 vs. 4.4, p=0.012). PACU stay and time to discharge were longer in the block group than non-block group (3.3 vs. 2.6 hours, p=0.004 and 3.1 vs. 2.9 days, p=0.012, respectively). However, the block group ambulated a significantly longer distance than the non-block group on the first postoperative day (median: 181.1 m vs. 73.2 m, p<0.001). The block group more often received acetaminophen and/or aspirin and gabapentinoids than the non-block group (94.5% vs. 75.0%, p<0.001 and 84.8% vs. 62.0%, p<0.001, respectively).

Conclusion: Both CESPB and CSAPB are effective regional anesthesia techniques for VATS postoperative pain management with clear clinical benefits over no regional anesthesia. A direct comparison of the analgesic efficacy of CESPB versus CSAPB indicated that CESPB is more effective than CSAPB in terms of pain control. These findings are consistent with existing literature and most recent practice recommendations.

## Introduction

Video-assisted thoracoscopic surgery (VATS) has progressively become the preferred surgical technique to diagnose and treat a variety of thoracic clinical conditions, including valvular and arterial disease, arterial coronary disease, non-cardiac vascular disease, and lung cancer. VATS is a minimally invasive approach that displays multiple benefits over traditional open thoracotomy, including decreased surgery time as well as reduced postoperative pain and opioid use, promoting enhanced recovery [1].

Despite critical advances in the surgical process following the introduction of VATS, patients continue to experience significant postoperative pain. Optimal pain management in the postoperative period is vital to promote patient comfort and accelerate recovery while minimizing the incidence of common pulmonary complications such as pneumonia and atelectasis [2,3]. To manage pain and reduce reliance on traditional opioid medications, multimodal analgesic care has become standard perioperative practice for many surgeries, including VATS [4]. In addition to non-opioid medications, regional nerve blocks can help achieve greater pain control, fostering efficient recovery and increased patient satisfaction.

The serratus anterior plane block (SAPB) represents one type of regional anesthesia technique used perioperatively for VATS. A local anesthetic is injected within the serratus anterior plane, providing analgesia to the hemithorax by blocking the lateral cutaneous branches of the intercostal nerves [5]. The SAPB quickly gained favor in clinical practice, where it has been described as technically less challenging to perform with a more favorable safety profile than other regional anesthetic techniques such as thoracic paravertebral and central neuraxial blockade [6]. The efficacy of SAPB in the setting of VATS has been well established; several randomized controlled trials reported decreased pain and opioid consumption when SAPB is used perioperatively compared to standard pain management alone [5,7,8].

The erector spinae plane block (ESPB) is a second type of regional anesthesia used to manage VATS postoperative pain; local anesthetic is administered via the dorsal and ventral rami of the thoracic spinal nerves [9]. Similar to SAPB, ESPB quickly gained popularity clinically and was found to be effective for pain management following a variety of thoracic surgeries [10]. In 2020, an RCT assessed the efficacy of ESPB in 76 patients who underwent VATS and reported that a single ESPB block markedly reduced acute postoperative pain, total opioid consumption, and time to discharge from the post-anesthesia care unit (PACU) compared to standard of care alone [11]. These findings are consistent with other studies that evaluated the efficacy of ESPB for pain and recovery management following thoracic surgeries [12].

It is clear that both regional nerve blocks are effective for postoperative pain management and promotion of enhanced recovery following VATS. Subsequent RCTs have more recently reported the superiority of ESPB over SAPB when directly comparing acute pain and other recovery outcomes in patients who underwent VATS [13-16]. However, a common limitation reported in these studies is the lack of comparison of these interventions when continuous infusion with a catheter is used rather than a single-injection block. At our institution, patients who underwent VATS postoperatively received either a continuous SAPB (CSAPB) or continuous ESPB (CESPB) as part of multimodal analgesic care. Therefore, we retrospectively compared the effectiveness of these blocks in VATS to enhance what’s currently known.

The purpose of the study was to compare the postoperative outcomes of patients who underwent VATS and received a CSAPB versus those who received a CESPB. This study additionally sought to compare the postoperative outcomes of patients who underwent VATS and received either regional anesthesia technique versus those who did not receive any regional analgesia. We hypothesized that the use of regional anesthesia for VATS, compared to no regional anesthesia, provided better analgesia, reducing opioid consumption, complications, and hospital stay. We additionally hypothesized that CESPB, compared to CSAPB, provided better postoperative analgesia, reducing postoperative opioid requirements, complications, and hospital stay.

This article was presented previously as a poster at the 2023 PGA PostGraduate Assembly in Anesthesiology Meeting on December 9, 2023.

## Materials and methods

Study design

This single-site, retrospective study compared several analgesic and recovery outcomes in patients who underwent VATS and received postoperative CESPB from November 2019 to October 2020 (CESPB group) to those who received postoperative CSAPB from September 2018 to October 2019 (CSAPB group). Patients receiving either of these blocks were then collated to form a single group (block) and compared to patients who did not receive any regional anesthesia from October 2017 to October 2018 (no block). This study was approved by the Hartford HealthCare Institutional Review Board (IRB; approval number: HHC-2021-0348). Patient data were retrieved through a retrospective review of the electronic medical record; no data were collected prospectively. Patients provided informed consent for VATS surgery and regional anesthesia. Since the study involved only minimal risk to the participants, a total waiver of written informed consent was granted by the IRB. The study reports followed the Strengthening the Reporting of Observational Studies in Epidemiology (STROBE) guidelines.

Study population

**Figure 1:**
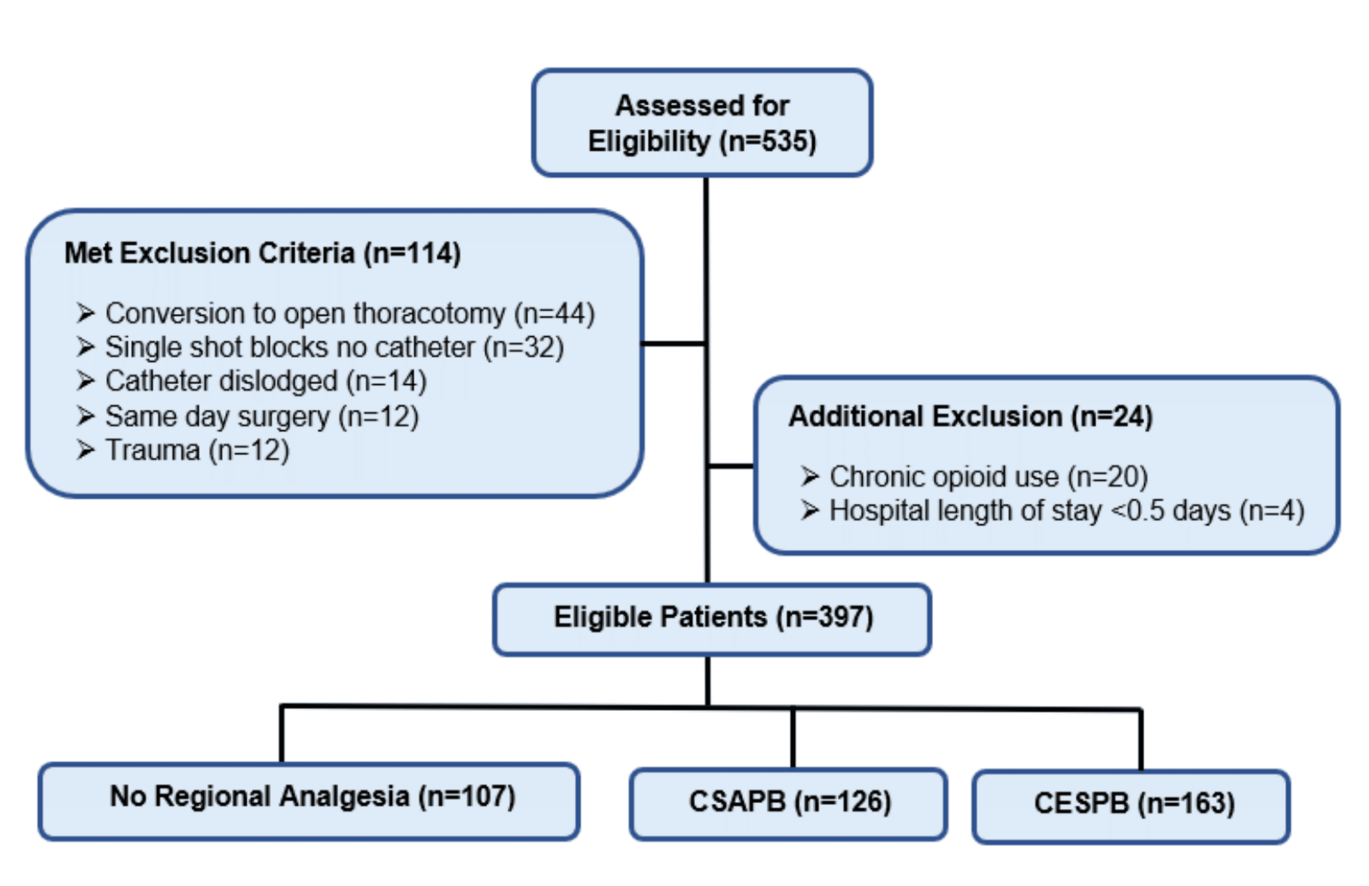
Flowchart of study patients CSAPB: continuous serratus anterior plane block, CESPB: continuous erector spinae plane block

Adult patients (≥18 years) who underwent VATS between October 2017 and October 2020 and received multimodal analgesia for postoperative pain control were included (Figure 1). The patients were divided into three study groups (CESPB, CSAPB, and no block) in accordance with their VATS surgery date and which continuous regional nerve block was given, if any. Patients who received regional anesthesia other than a CESPB or CSAPB during the study period (e.g., single injection SAPB/ESPB without catheter placement or other block type) were excluded from the study. Any patients with an incidence of block catheter dislodgement or removal prior to the planned date were additionally excluded. Other exclusion criteria included VATS cases that converted to open thoracotomy, VATS performed on patients with trauma, and VATS performed as same-day surgeries. Patients with chronic opioid use, defined as continuous use of opioids for 60 or more days prior to surgery, and those with a hospital length of stay of less than half a day were also excluded.

Study objectives and data collection

All data were retrospectively obtained from patient EMRs using the hospital’s Electronic Privacy Information Center (EPIC). To ensure comparability of the study groups and account for potential confounding variables, patient demographics (age, gender, race, and ethnicity), health characteristics (body mass index (BMI) and American Society of Anesthesiology (ASA) score), and comorbidities at the time of VATS were obtained. The Charlson Comorbidity Index (CCI) with Quan modification was also used to summarize patient comorbidity burden [17]. Age adjustment was applied (<50 years = 0, 50-59 = 1, 60-69 = 2, 70-79 = 3, ≥80 = 4), and weighted CCI scores were stratified by total score (none = 0, mild = 1-2, moderate = 3-4, and severe = ≥5). All anesthesia and surgical records during VATS hospital admission were carefully reviewed for each patient using EPIC to verify patient eligibility in the study. The use of multimodal analgesia (MMA) during the VATS in-patient period was confirmed for each patient via medication records in EPIC; MMA comprised two or more drugs and/or interventions not including systemic opioids that act by different mechanisms to provide analgesia; these drugs can be administered by the same or different routes and included non-steroidal anti-inflammatory drugs, muscle relaxants, gabapentinoids, and local anesthetics for regional block administration.

Co-primary Objectives

A co-primary objective of this study was to determine if the use of CESPB after VATS reduces opioid consumption compared to that of those who received CSAPB. To calculate total opioid consumption during the patients’ hospital stays, all opioid analgesic types, doses, and routes of administration were collected, sorted in accordance with time given (preoperative, intraoperative, and postoperative), and presented as morphine milligram equivalent (MME). A second co-primary objective sought to compare the impact of these blocks on the quality of pain control in the first 72 postoperative hours. Minimum, maximum, and average postoperative pain scores at rest and during activity were compared using the numeric pain scale (NPS). To evaluate the quality of postoperative analgesia provided by either regional block, patients that received either CESPB or CSAPB were collated to form the block group and compared to a no block group for the aforementioned co-primary outcomes and all secondary outcomes described.

Secondary Objectives

To compare the analgesic efficacy of CESPB versus CSAPB postoperatively for VATS, several other outcomes were included. PACU length of stay, time to first opioid medication, and time from procedure end to hospital discharge were documented from EPIC. The median distance ambulated on the first postoperative day was obtained from inpatient physical therapy progress notes to gauge the impact of each block on recovery. Inpatient progress notes from anesthesiology, physical therapy, and other care providers were used to determine the incidence of VATS postoperative complications, including infection, respiratory diagnoses, postoperative nausea and/or vomiting (PONV), and regional block-related adverse events. Regional block-related adverse events were defined as any infection, bleeding or hematoma, neuropathy, local anesthetic systemic toxicity, or pneumothorax. To assess for PONV incidence in each study group, antiemetics use during the VATS inpatient period was collected and reported as the number and frequency of patients that received any antiemetic, including ondansetron, dronabinol, dexamethasone, metoclopramide, or meclizine. To verify all patients received MMA and account for the influence of multimodal analgesic care following VATS on study outcomes, a breakdown of non-opioid analgesic use was reported among the study groups.

Statistical Analysis

A preliminary analysis was conducted to evaluate the underlying distribution of numerical data for the normality test. Binary and categorical information were described with frequencies and percentages; either mean and standard deviation or median and interquartile range (IQR) were reported for continuous data based on the underlying distribution. For two-group comparisons, Mann-Whitney U tests or T-tests were used for numerical information, while Pearson Chi-Square tests were conducted for binary or categorical data. Fisher's Exact Tests were applied for binary variables with small samples in cells.

The log-transformed total MME was generated in preparation for multivariate statistics, indicating six outliers should be eliminated (two no block, three CSAPB, and one CESPB) for multivariate regressions. Variables with a p≤0.1 in univariate analysis were included for multivariate regressions with a stepwise selection approach. Stepwise linear regressions were used for log-transformed total MME, average pain score at rest, and average pain score during activity. A backward stepwise (Wald) logistic regression was applied for greater than or equal to six days of hospital stay. All statistical results yielding a p-value <0.05 were considered statistically significant; all analyses were carried out using SPSS Statistics version 29 (IBM Corp. Released 2023. IBM SPSS Statistics for Windows, Version 29.0.2.0 Armonk, NY: IBM Corp).

## Results

Of 535 patients who underwent VATS at our institution between October 2017 and October 2020, a total of 397 patients met the inclusion criteria for this study. Of these patients, 108 (27.2%) did not receive regional analgesia, and 289 (72.8%) received either CSAPB (n=126, 43.6%) or CESPB (n=163, 56.4%). There were no significant differences in the baseline characteristics of patients receiving CSAPB versus those receiving CESPB (Table 1). However, when comparing the baseline characteristics of patients who received regional analgesia (CSAPB or CESPB, block group) versus those who did not (non-block group), a greater proportion of the block group exhibited moderate comorbidity burden (p=0.001). The block group also had a shorter pre-surgical hospital stay than the non-block group (p=0.023).

**Table 1:** Patient demographics and clinical characteristics The data is presented as median and IQR or number and frequency (%). A p-value less than 0.05 is considered statistically significant. Mann-Whitney U Test or T-test is used for numerical data, Pearson Chi-Square statistics is used for binary categorical information, and Fisher’s Exact Test is used for binary variables and small sample sets. †: ethnicity is missing for three patients, race is missing for four patients, and ASA is missing for one patient. CSAPB: continuous serratus anterior plane block, CESPB: continuous erector spinae plane block, IQR: interquartile range, BMI: body mass index, ASA: American Society of Anesthesiologists, CCI: Charlson Comorbidity Index

Characteristic	By regional block			No block vs. block		
	CSAPB (n=126)	CESPB (n=163)	p-value	No block (n=108)	Block (n=289)	p-value
Age, median (IQR)	67 (58-72)	65 (57-73)	0.948	64 (54-74)	65 (57-73)	0.797
BMI, kg/m^2^, median (IQR)	27.0 (23.3-30.9)	27.4 (22.6-31.8)	0.676	27.1 (23.9-31.6)	27.3 (22.9-31.7)	0.860
Sex, n (%)			0.599			0.086
Female	75 (59.5)	92 (56.4)		52 (48.1)	167 (57.8)	
Male	51 (40.5)	71 (43.6)		56 (51.9)	122 (42.2)	
Ethnicity, n (%)^†^			0.764			0.905
Hispanic or Latino	8 (6.4)	9 (5.6)		6 (5.6)	17 (5.9)	
Not Hispanic or Latino	117 (93.6)	153 (94.4)		101 (94.4)	271 (94.1)	
Race, n (%)^†^			0.840			0.685
White or Caucasian	111 (88.8)	138 (86.3)		91 (84.3)	249 (87.4)	
Black or African American	5 (4.0)	10 (6.3)		5 (4.6)	15 (5.3)	
Asian	2 (1.6)	2 (1.3)		2 (1.9)	4 (1.4)	
Other	7 (5.6)	10 (6.3)		10 (9.3)	17 (6.0)	
ASA physical status, n (%)^†^			0.638			0.219
1	0 (0.0)	1 (0.6)		2 (1.9)	1 (0.3)	
2	25 (19.8)	25 (15.4)		20 (18.5)	50 (17.4)	
3	95 (75.4)	128 (79.0)		77 (71.3)	223 (77.4)	
4	6 (4.8)	8 (4.9)		9 (8.3)	14 (4.9)	
5	0 (0.0)	0 (0.0)		0 (0.0)	0 (0.0)	
CCI, age-adjusted, n (%)			0.651			0.001
1. None (0)	36 (28.6)	42 (25.8)		48 (44.4)	78 (27.0)	
2. Mild (1-2)	59 (46.8)	70 (42.9)		44 (40.7)	129 (44.6)	
3. Moderate (3-4)	21 (16.7)	36 (22.1)		7 (6.5)	57 (19.7)	
4. Severe (≥5)	10 (7.9)	15 (9.2)		9 (8.3)	25 (8.7)	
Pre-surgery hospital stay, median (IQR)	0.2 (0.2-0.3)	0.2 (0.2-0.4)	0.056	0.3 (0.2-1.2)	0.2 (0.2-0.3)	0.023

Co-primary outcomes

In the first 72 postoperative hours, patients who received CESPB reported lower minimum and average pain with activity than those who received CSAPB (p=0.004 and p=0.009, respectively), whereas pain at rest was statistically similar (Table 2). Median total MME opioid consumption with IQR was similar in the two study groups (CSAPB: 90 (36-180) vs. CESPB: 87 (45-182), p=0.539) and remained similar when stratified according to time given. Considering both regional blocks versus no block, the block group reported lower minimum and average pain at rest and with activity in the first 72 postoperative hours (rest: p=0.004 and p=0.012; activity: p<0.001 and p=0.012; respectively). Median total opioid consumption was statistically similar between the block and non-block groups; however, the block group was given fewer intraoperative opioids than the non-block group (median MME (IQR): block: 18 (14-26) vs. no block: 26 (17-33), p<0.001).

**Table 2:** Co-primary outcomes The data is presented as median and IQR or mean and SD. A p-value less than 0.05 is considered statistically significant. Mann-Whitney U Test or T-test is used for numerical data. CSAPB: continuous serratus anterior plane block, CESPB: continuous erector spinae plane block, NPS: numeric pain scale, IQR: interquartile range, SD: standard deviation, MME: morphine milligram equivalent

Outcome	By regional block			No block vs. block		
	CSAPB (n=126)	CESPB (n=163)	p-value	No block (n=108)	Block (n=289)	p-value
Pain (NPS, first 72 postoperative hours)						
At rest						
Minimum, median (IQR)	0.0 (0.0-0.0)	0.0 (0.0-0.0)	0.107	0.0 (0.0-0.0)	0.0 (0.0-0.0)	0.004
Maximum, median (IQR)	8.0 (7.0-10.0)	8.0 (7.0-10.0)	0.709	8.0 (6.3-9.0)	8.0 (7.0-10.0)	0.162
Average, mean (SD)	3.7 (1.7)	3.4 (1.6)	0.114	4.1 (1.6)	3.6 (1.7)	0.012
With activity						
Minimum, median (IQR)	0.0 (0.0-0.0)	0.0 (0.0-0.0)	0.004	0.0 (0.0-2.0)	0.0 (0.0-0.0)	<0.001
Maximum, median (IQR)	8.0 (6.0-10.0)	8.0 (6.0-10.0)	0.527	8.0 (6.0-9.0)	8.0 (6.0-10.0)	0.328
Average, mean (SD)	4.2 (1.9)	3.6 (1.8)	0.009	4.4 (1.9)	3.8 (1.8)	0.012
Opioid consumption (MME), median (IQR)						
Preoperative	0 (0-0)	0 (0-0)	0.325	0 (0-0)	0 (0-0)	0.051
Intraoperative	17 (13-26)	18 (15-26)	0.551	26 (17-33)	18 (14-26)	<0.001
Postoperative	74 (20-140)	58 (23-149)	0.914	72 (29-139)	64 (22-148)	0.438
Total	90 (36-180)	87 (45-182)	0.539	108 (58-181)	88 (43-181)	0.115

Secondary outcomes

Time to first postoperative opioid, PACU length of stay, and time to discharge were all similar among patients who received CESPB or CSAPB (Table 3). When grouped together, the block group stayed in the PACU and hospital longer than the non-block group following VATS (p=0.004 and p=0.012, respectively). No differences were observed in ambulation distance on postoperative day one between patients who had received CESPB versus CSAPB; taken together and compared to the non-block group, the block group ambulated over double the distance on postoperative day one (p<0.001).

**Table 3:** Secondary outcomes The data is presented as median and IQR or number and frequency (%). A p-value less than 0.05 is considered statistically significant. a: Mann-Whitney U Test is used for numerical data, Pearson Chi-Square statistics is used for binary categorical information, and Fisher’s Exact Test is used for binary variables and small sample sets. b: N=283 (71.3%) CSAPB: continuous serratus anterior plane block, CESPB: continuous erector spinae plane block, IQR: interquartile range, NSAIDs: non-steroidal anti-inflammatory drugs, PACU: post-anesthesia care unit, PONV: postoperative nausea and/or vomiting

Outcome	By regional block			No block vs. block		
	CSAPB (n=126)	CESPB (n=163)	p-value^a^	No block (n=108)	Block (n=289)	p-value^a^
Time to first opioid after surgery, median minutes (IQR)	36.0 (8.0-117.0)	25.5 (11.0-55.0)	0.346	22.0 (8.0-53.7)	28.5 (10.0-84.5)	0.225
PACU length of stay, median hours (IQR)	3.0 (2.2-4.6)	3.4 (2.3-4.7)	0.328	2.6 (2.0-4.4)	3.3 (2.3-4.7)	0.004
Procedure end to discharge, median days (IQR)	3.0 (2.1-4.0)	3.1 (2.1-5.0)	0.058	2.9 (1.9-4.1)	3.1 (2.1-4.3)	0.012
Ambulation distance, median meters (IQR)^b^	130.2 (48.8-351.4)	222.5 (84.3-374.3)	0.089	73.2 (45.7-121.9)	181.1 (65.5-369.4)	<0.001
Pain medications, n (%)						
Acetaminophen or aspirin	122 (96.8)	151 (92.6)	0.123	81 (75.0)	273 (94.5)	<0.001
Other NSAIDs	57 (45.2)	59 (36.2)	0.120	46 (42.6)	116 (40.1)	0.658
Musculoskeletal relaxants	26 (20.6)	48 (29.4)	0.089	22 (20.4)	74 (25.6)	0.278
Gabapentin or pregabalin	104 (82.5)	141 (86.5)	0.352	67 (62.0)	245 (84.8)	<0.001
Adverse events, n (%)						
Respiratory	1 (0.8)	3 (1.8)	0.635	0 (0.0)	4 (1.4)	0.578
Infection	0 (0.0)	1 (0.6)	1.000	0 (0.0)	1 (0.3)	1.000
PONV	2 (1.6)	1 (0.6)	0.582	0 (0.0)	3 (1.0)	0.566
Regional (block-related)	8 (6.3)	13 (8.0)	0.597	-	21 (7.3)	
Antiemetics, n (%)	35 (27.8)	41 (25.2)	0.615	29 (26.9)	76 (26.3)	0.911

All patients received MMA; no differences were observed in the pain regimen provided to patients who received CESPB versus CSAPB. When collated and compared to the non-block group, the block group received acetaminophen and/or aspirin as well as gabapentin and/or pregabalin more often (p<0.001, both). No significant differences in VATS postoperative complications were observed between study groups. The incidence of block complications overall was about 7.3% (21 of 289), and complication rates were similar between the CESPB and CSAPB groups (13 (8.0%) vs. 8 (6.3%), p=0.597). Antiemetics use was similar across all study groups.

Statistical modeling

Log transformation of the total opioid consumption data for all 397 patients revealed six outliers; thus, 391 patient records were included further by multivariate regression with a stepwise approach. Table 4 summarizes essential predictors of the co-primary outcomes, total opioid consumption, and 72-hour postoperative pain. Regression analyses indicated that total opioid consumption and pain were positively associated with preoperative and intraoperative opioid consumption as well as musculoskeletal relaxant use (p=0.001 or p<0.001, all). Conversely, male sex (p=0.013 and p=0.014), management with a CESPB (p=0.003 and p<0.001), and lower pre- and intraoperative opioid consumption (p=0.001 and p<0.001, p=0.003 and p<0.001, respectively) were associated with lower average pain at rest and during activity.

**Table 4:** Multivariate linear regression for log-transformed total opioid consumption (MME) and average pain (both at rest and with activity) The data is presented as beta and 95% CI. A p-value less than 0.05 is considered statistically significant. a: model indexes: adjusted R2=0.348, Durbin-Watson=0.453, b: model indexes: adjusted R2=0.155, Durbin-Watson=1.479, c: model indexes: adjusted R2=0.144, Durbin-Watson=1.402 MME: morphine milligram equivalent, CI: confidence interval, CESPB: continuous erector spinae plane block, CI: confidence interval

Associated variables	Beta (95% CI)	p-value
Opioid consumption (MME)^a^		
Preoperative opioid consumption (MME)	0.0 (0.0-0.0)	<0.001
Intraoperative opioid consumption (MME)	0.0 (0.0-0.0)	<0.001
Musculoskeletal relaxants	0.7 (0.5-0.9)	<0.001
Average pain at rest^b^		
Male sex	-0.4 (-0.7- -0.1)	0.013
Management with CESPB	-0.5 (-0.8- -0.2)	0.003
Preoperative opioid consumption (MME)	0.9 (0.6-1.3)	<0.001
Intraoperative opioid consumption (MME)	0.0 (0.0-0.0)	0.001
Musculoskeletal relaxants	0.9 (0.6-1.3)	<0.001
Average pain with activity^c^		
Male sex	-0.4 (-0.8- -0.1)	0.014
CESPB	-0.7 (-1.0- -0.3)	<0.001
Preoperative opioid consumption (MME)	0.9 (0.5-1.3)	<0.001
Intraoperative opioid consumption (MME)	0.0 (0.0-0.0)	<0.001
Musculoskeletal relaxants	0.9 (0.5-1.3)	<0.001

Table 5 summarizes the results of multivariate logistic regression for extended hospital stay (greater than or equal to six days). Both regional blocks were associated with a higher risk of prolonged hospital stay, though only CESPB was statistically significant (p=0.001); an extended pre-surgery hospital stay (p=0.003) and musculoskeletal relaxant use (p=0.004) were also associated with a higher risk of prolonged stay. Preoperative opioid consumption did not affect the risk of prolonged hospital length of stay, while the use of gabapentinoids was associated with a lower risk of prolonged hospital stay (p=0.006).

**Table 5:** Multivariate logistic regression for ≥6 days of hospital stay The data is presented as OR and 95% CI. A p-value less than 0.05 is considered statistically significant. Model indexes: Nagelkerke R2=0.156, Hosmer and Lemeshow Test=0.923, area under the ROC curve=0.73 OR: odds ratio, CI: confidence interval, CSAPB: continuous serratus anterior plane block, CESPB: continuous erector spinae plane block, MME: morphine milligram equivalent

Associated variables	OR (95% CI)	p-value
Reference group (no regional block)		0.004
Management with CSAPB	2.5 (0.9-6.7)	0.076
Management with CESPB	4.6 (1.8-11.5)	0.001
Musculoskeletal relaxants	2.6 (1.4-5.1)	0.004
Gabapentin or pregabalin	0.4 (0.2-0.7)	0.006
Pre-surgery hospital stay (days)	1.2 (1.1-1.4)	0.003
Preoperative opioid consumption (MME)	1.0 (1.0-1.0)	0.058

## Discussion

The use of a regional anesthetic technique as part of multimodal analgesic care is strongly recommended for VATS unless contraindicated [18]. Multiple clinical research studies have demonstrated the benefits of regional anesthesia for VATS pain management and recovery, among which the ESPB and SAPB are frequently cited [5,7,8,11,13-16]. Recent PROSPECT guidelines for VATS specifically recommend first-line use of either a paravertebral or ESPB and the use of a SAPB as a second-line option [18]. Yet, few studies have directly compared the efficacy of ESPB to SAPB for VATS using continuous infusion rather than a single-injection approach. The use of continuous infusion via a catheter can prolong analgesia for several days compared to a single-injection block [19], underscoring the value of comparing the analgesic quality of these blocks when a continuous approach is used. In this retrospective chart analysis, two analyses were conducted. One analysis evaluated the efficacy of the CESPB and CSAPB as a collated regional "block" group compared to historical "non-block" controls; a second analysis reflected the study’s primary intention to compare the analgesic efficacy of CESPB to CSAPB following VATS.

The use of either continuous regional block was associated with significant improvements in VATS postoperative outcomes, specifically reduced pain and greater mobilization. These results align with prior studies that report multiple benefits of either CESPB or CSAPB for postoperative pain management and VATS recovery [20-24]. Though the block group remained in the PACU and hospital slightly longer than the non-block group (3.3 vs. 2.6 hours and 74.4 vs. 69.6 hours, respectively) and had a higher risk of prolonged hospital stay, these differences were small and unlikely to be attributable to the blocks. At baseline, the block group exhibited significantly greater comorbidity burden and reduced time to VATS, likely reflecting more complex cases with increased surgical urgency, necessitating a longer length of stay before transfer and discharge clearance.

Though regional anesthesia is recommended for VATS regardless of comorbidity status, regional anesthesia was favored for patients suffering from mild or moderate comorbidity burden. This finding is reasonable given patients with comorbidities retain a higher risk for VATS complications [25], and enhanced recovery after surgery guidelines specifically recommend regional anesthesia to help prevent such complications [26]. This plausibly explains why the block group demonstrated heightened comorbidity compared to historical non-block controls. This difference in baseline health provides evidence to support the use of regional anesthesia in VATS given that the block group reported less pain and mobilized more than the non-block group despite greater comorbidity. Overall, the study findings concur with existing literature and current guidelines supporting the use of both regional anesthesia techniques for VATS postoperative management.

At our institution, CSAPB and CESPB have been consecutively used in VATS postoperative management as part of a multimodal analgesic care regime, allowing for retrospective comparison of these anesthesia techniques. In line with previous literature [13-15,27], patients who received CESPB reported significantly lower pain with activity; though not statistically significant, pain at rest was also lower in the CESPB group. In terms of opioid use, the CSAPB and CESPB patient groups were comparable. Finnerty et al. also reported no difference in opioid consumption [14], though several other trials do report lower opioid use with ESPB, and the time to first analgesic was prolonged with ESPB in all related trials [13,15,27]. Risk factors for heightened postsurgical pain and opioid use following VATS included female sex and presurgical opioid use, both of which are consistent with existing literature [28]. Incorporation of regional anesthesia and multimodal analgesics in VATS postoperative care was associated with reduced risk of enhanced pain, especially when a CESPB and gabapentinoids were used. Taken together, these findings suggest analgesic superiority of CESPB over CSAPB, in line with the most recent VATS PROSPECT practice guideline and coinciding with our institution’s historical transition from SAPB to ESPB in standard practice.

The main limitation of this study is the difference in time periods from which the study groups were compared due to its retrospective nature. Increased use of multimodal analgesia and favor of regional anesthesia among patients with heightened comorbidity represent distinct differences between the non-block and block groups and reflect widespread efforts to optimize recovery and prevent postoperative complications among patients who undergo VATS. Despite the difference in time periods evaluated, baseline health and multimodal analgesia use were quite similar between the CESPB and CSAPB groups, allowing for a more reliable comparison. Specific administration details such as infusion rates, adjuvants used, and length of catheter use were not compared between these block groups due to heterogeneity in the course of care among patients in the study. However, all regional anesthesia blocks were administered based on the standard practice guidelines, using weight-based doses of either bupivacaine or ropivacaine local anesthetics, and performed under ultrasound guidance.

## Conclusions

Postoperative regional anesthesia is a highly beneficial clinical practice to mitigate pain and optimize recovery following VATS. Several regional anesthesia techniques have been used for pain management following VATS, including CESPB or CSAPB.

In this study, both blocks demonstrated clinical benefits; however, overall, the ESPB demonstrated analgesic superiority over the SAPB. These findings support the preferred and continued use of the ESPB for VATS postoperative management in line with current clinical practice guidelines. Future studies may benefit from weighing the benefits and risks of postoperative continuous versus single injection applications of the ESPB in the VATS setting.
